# The perceived facilitators and barriers to the potential adoption of teledentistry among dental care providers in Saudi Arabia: A systematic review

**DOI:** 10.1371/journal.pone.0359436

**Published:** 2026-09-28

**Authors:** Amjad Hakami, Sarika Mohan

**Affiliations:** Department of Health Data Science, Division of Health Informatics, Swansea University, Swansea, Wales, United Kingdom; King Faisal University, SAUDI ARABIA

## Abstract

Teledentistry has emerged as a tool that holds great potential to enhance access, efficiency, and continuity of oral healthcare delivery. In Saudi Arabia, its adoption is important in the context of the health transformation programme and Vision 2030, particularly given the high prevalence of dental caries among schoolchildren. Despite this potential, actual implementation is still limited. In this context, there is a need to systematically assess the factors influencing its potential adoption from the care providers’ perspectives. This review aimed to systematically identify how dental care providers perceive the facilitators and barriers to the adoption of teledentistry in Saudi Arabia, with a focus on the perception of dental care providers, including dental students. Following PRISMA 2020 and SWiM guidelines, PubMed, Scopus, Medline, Embase, and CINAHL databases were searched, covering publications up to 4 July 2025. The PICO framework was used to define the inclusion and exclusion criteria, and the CASP checklist was used to assess study quality. Data were synthesised using vote counting by direction of effect, categorising findings into facilitators and barriers. Out of 156 records, 11 studies met the inclusion criteria. Study quality ranged from 7 to11 out of 11 based on the CASP checklist for cross-sectional studies. Key findings included potential facilitators such as improved access, efficiency and cost-effectiveness, communication and referral enhancements, educational benefits, and positive perception of technology. The key reported barriers were limited awareness and training, infrastructure and equipment reliability, privacy and data security, cost and financial issues, diagnostic accuracy, and the preference for in-person meetings. While teledentistry holds great potential, its integration in Saudi Arabia could be hindered by educational and technical challenges. Addressing them through improved infrastructure, enhanced training and integration into dental curricula, and strengthened regulatory frameworks may facilitate and support equitable adoption.

## Introduction and background

Digital health transformation has introduced new technologies that can enhance healthcare delivery, one of which is telemedicine [1]. Teledentistry, as a branch of telemedicine, refers to the use of information and communication technologies (ICTs) to deliver dental care, consultation, advice, and treatment in a cost-effective and accessible manner, thereby enhancing accessibility for underserved populations [2]. The first appearance of this tool was in 1994 as a military project in the United States, which aimed to provide patient care, continuous dental education, and to facilitate communication with dental laboratories [3]. Its applications include referrals to specialists, patient consultations, educational benefits, and monitoring patients remotely [2].

In Saudi Arabia, the prevalence of dental caries remains alarmingly high, especially among schoolchildren, which requires massive efforts to reduce its burden and improve oral health outcomes [4]. Utilising technological advancements such as teledentistry can help address this issue, support screening and oral health promotion, and foster interprofessional collaboration [5].

Moreover, as part of the health sector transformation programme, the utilisation of ICTs is a key strategy to enhance both accessibility and quality of care [6]. Within this context, teledentistry is considered a promising tool to expand access, improve care quality, and support the objectives of Saudi Vision 2030 [6,7].

Despite the growing global interest in teledentistry, its integration into routine dental practice remains inconsistent due to persistent barriers related to infrastructure, training, and regulatory frameworks, among others [8]. Understanding dental care providers’ views and perceptions of these tools and applications is essential to support potentially effective adoption [9]. In Saudi Arabia, several cross-sectional studies have explored the awareness, attitudes, and perceptions of dental care providers; however, these studies are fragmented and vary in their methodological approaches and findings. To date, there is a lack of systematic synthesis evaluating the facilitators and barriers influencing the adoption of teledentistry among dental care providers in Saudi Arabia. Therefore, this systematic review aims to synthesise the available evidence on the potential facilitators and barriers to the adoption of teledentistry among dental care providers in Saudi Arabia and assess their overall readiness to integrate these digital tools into routine dental practice, which can provide insights to guide future implementation strategies and evidence-based policies that align with Saudi Vision 2030.

## Materials and methods

### Study design

This study is a systematic review of quantitative cross-sectional studies, applying the Synthesis Without Meta-analysis (SWiM) guidelines and summarised narratively [10,11]. The review was conducted in accordance with the Preferred Reporting Items for Systematic Reviews and Meta-Analyses (PRISMA) 2020 guidelines [12] (S1Checklist). This review was registered with the International Prospective Register of Systematic Reviews (PROSPERO), registration number: CRD420251145570.

### Ethical considerations

Given that this study is a systematic review of previously published studies, no new data were collected, all data were extracted from publicly available literature, and formal ethical approval was not required [13].

### Search strategy

A comprehensive search strategy was applied to identify all relevant publications up to 4 July 2025. The terms “Teledentistry” AND “Saudi Arabia” were used in the Medline, Embase, PubMed, Scopus, and CINAHL databases. Equivalent MeSH terms and free-text keywords were included to ensure a sensitive search (e.g., “Teledentistry”, “Teledental”, “Saudi Arabia”, “KSA”). Broader terms such as “telehealth” or “telemedicine” were excluded to maintain search specificity and prevent the retrieval of non-dental studies. S1 Table shows full database search terms.

### Eligibility criteria

The inclusion and exclusion criteria were defined using the PICO framework [14,15], where the population comprised dental care providers in Saudi Arabia (dentists and dental students), the intervention was the potential use of any form of teledentistry, with no comparison, and the outcomes were quantitative measures of the potential facilitators and barriers to the adoption of teledentistry (S2 Table).

Only studies that reported quantitative findings were included, as the review aimed to synthesise measurable outcomes of the potential facilitators and barriers to teledentistry adoption. Qualitative-only studies, studies conducted outside of Saudi Arabia, and studies that examined patients’ perspectives were excluded to strictly focus on healthcare providers’ readiness. The full inclusion and exclusion criteria are available in S3 Table.

### Study selection process

The database search produced 156 records, of which 90 duplicates were removed. A total of 66 titles and abstracts were screened, resulting in the exclusion of 35 records, due to an irrelevant population, study design, or outcomes. The remaining 31 full-text articles were assessed for eligibility, and 11 studies met the inclusion criteria (Fig 1).

**Fig 1:**
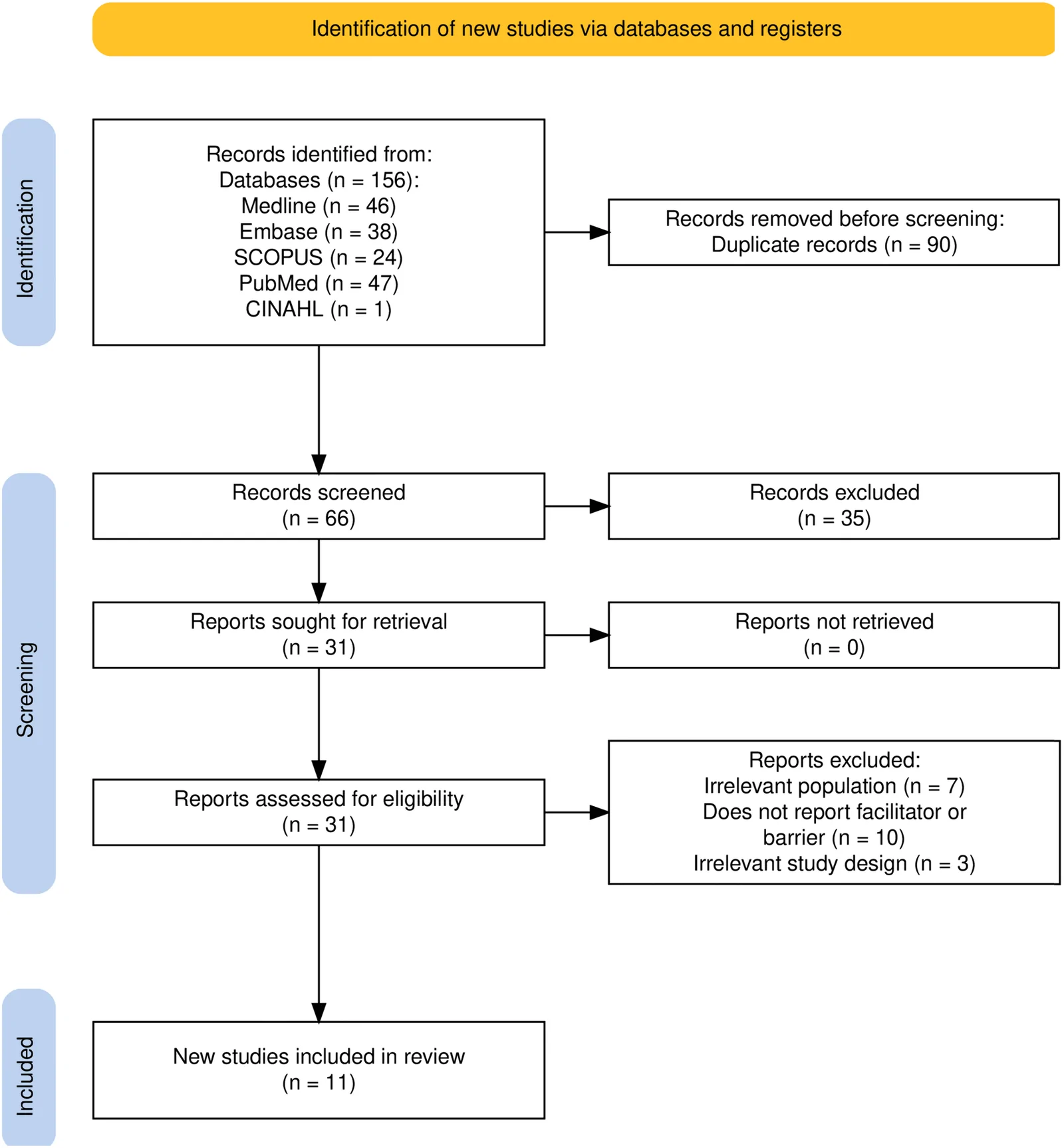
PRISMA flow diagram.

All included studies were cross-sectional surveys involving a total of 5,739 participants, comprising both dental practitioners and dental students across Saudi Arabia. Sample sizes ranged from 148 to 2,143 participants.

### Quality appraisal

Quality appraisal was conducted to enhance the rigour and strength of the evidence [16]. The methodological quality of the included studies was appraised using the Critical Appraisal Skills Programme (CASP) checklist for cross-sectional studies [17]. The CASP tool was used because it provides a transparent framework that assesses aspects of cross-sectional survey methodology, including recruitment strategy, potential measurement and selection bias, statistical rigor, and applicability of findings. The overall quality scores ranged from 7 to 11 out of 11 indicating moderate to high quality (Table 1). The scores were used to summarise the extent to which each study met the appraisal criteria and to inform interpretation of the strength of the evidence.

**Table 1:** Studies CASP scores.

Author (Year)	Clearly focused issue	Appropriate method	Acceptable recruitment way	Measures to reduce bias	Appropriate data collection method	Enough participants to reduce play of chance	Clear presentation of the results	Rigorous data analysis	Findings clearly stated	Results applicable to local population	Research is valuable	Score out of 11
Aboalshamat, 2020	Yes	Yes	Yes	Can’t tell	Yes	Yes	Yes	Can’t tell	Yes	Yes	Yes	9
Alawwad et al., 2019	Yes	Yes	Can’t tell	Can’t tell	Yes	Can’t tell	Yes	Can’t tell	Yes	Yes	Yes	7
Al-Khalifa & AlSheikh, 2020	Yes	Yes	Yes	Yes	Yes	Yes	Yes	Yes	Yes	Yes	Yes	11
Alshammari & Almaktoom, 2023	Yes	Yes	No	Can’t tell	Yes	Yes	Yes	Yes	Yes	Yes	Yes	9
Alsharif & Al-Harbi, 2020	Yes	Yes	No	No	Yes	Yes	Yes	Yes	Yes	Yes	Yes	9
Nassani et al., 2021	Yes	Yes	Yes	Yes	Yes	Yes	Yes	No	Yes	Can’t tell	Yes	9
Sharka et al., 2023	Yes	Yes	Can’t tell	Yes	Yes	Yes	Yes	Yes	Yes	No	Yes	9
Joseph et al., 2024	Yes	Yes	Yes	Yes	Yes	Yes	Yes	Yes	Yes	Yes	Yes	11
Aldhamen & Al Dosari, 2023	Yes	Yes	Can’t tell	No	Yes	Yes	Yes	Yes	Yes	No	Yes	8
Almazrooa et al., 2021	Yes	Yes	No	No	Yes	Can’t tell	Yes	Yes	Yes	Yes	Yes	8
Khan et al., 2022	Yes	Yes	No	No	Yes	Yes	Yes	Yes	Yes	Yes	Yes	9

### Data extraction

Based on the Cochrane Guidelines on Narrative Synthesis, data were extracted into a structured table that included study authors and year, population characteristics, study design, aim of the study, and key findings. Data were extracted and then reviewed again to ensure accuracy (S4 Table).

### Data synthesis

Due to heterogeneity in study designs, outcomes, and measurement tools, a narrative synthesis was adopted following the Cochrane Guide on Narrative Synthesis [18] and the SWiM guidelines [10]. As all included studies relied on self-administered questionnaires, the synthesis reflects participants’ self-perceptions of facilitators and barriers.

Percentage-based measures were used as the standardised metric to summarise the proportion of participants reporting barriers or facilitators, enabling consistent comparison across studies with diverse reporting formats. Perceived facilitators were grouped into five thematic groups and reported barriers into six (S5 and S6 Tables). The results were synthesised using vote counting based on the direction of effect rather than the effect size, as it was inconsistently reported, which does not account for study weights or sample size variations. It is important to emphasise that vote counting does not account for study precision, study weighting, or effect sizes; therefore, reported thematic frequencies should be interpreted cautiously as a qualitative synthesis of perceived determinants rather than quantitative evidence of the effect magnitude or relative importance.

For each theme, the number of studies, reported effect range, cumulative sample sizes, and CASP scores were extracted. The evidence strength of each theme was assessed by combining the average CASP score, number of studies, and cumulative sample size. This author-developed framework was designed to provide a systematic and transparent evaluation of evidence by considering study quality, quantity, and consistency. Evidence was rated considering all three criteria simultaneously as: High (Average CASP ≥ 9 out of 11, ≥ 5 independent studies, and cumulative sample size ≥ 2,000 participants); Moderate (Average CASP 7-8 out of 11, ≥ 3 studies, or cumulative sample size 1,000-1,999 participants); or Low (Average CASP ≤ 6 out of 11, < 3 studies, or cumulative sample size < 1,000 participants). A theme was downgraded to moderate if it failed to meet the sample size or study count threshold, even if average CASP score was ≥ 9. (Table 2). This provides a transparent indication of evidence strength but does not replace meta-analysis and should be cautiously interpreted given study heterogeneity [16].

## Results

S5 and S6 Tables show the thematic grouping of the perceived facilitators and reported barriers. Table 2 summarises the evidence strength for each theme across the studies.

### Facilitators

#### Improved access to care.

Several studies highlighted improved access as a major potential facilitator of teledentistry. Aboalshamat [19] reported that 52.2% of participants believed it would enhance access, and 60.8% thought it could facilitate easy and early specialist consultations. Similarly, a large proportion of respondents in other studies agreed that teledentistry has the potential to improve accessibility, especially for underserved or rural populations, with estimates ranging from 59.5% to 86.6% across six studies [20–25].

**Table 2:** Strength of evidence based on themes.

Theme	Number of Studies	Range (%)	Cumulative N	Strength of Evidence	Basis for Strength
**Perceived facilitators**					
Improved access to care	7	52.2–86.6	3,035	High	7 studies N = 3035, CASP 8–11 (Average 9/11)
Efficiency & cost-effectiveness	11	50.0–83	5,739	High	11 studies, N = 5,739, CASP 7–11 (Average 9/11)
Professional communication & referrals	4	55.0–79.5	1,279	Moderate	4 studies, N = 1,279, CASP 8–11 (Average 9/11)
Educational benefits	8	54.0–81.4	3,261	High	8 studies, N = 3,261, CASP 7–11 (Average 9/11)
Positive perception of technology	8	49.7–83.8	4,762	High	8 studies, N = 4,762, CASP 9–11 (Average 9/11)
**Reported barriers**					
Limited awareness & training	7	17.2–79.8	4,460	High	7 studies, N = 4,460, CASP 7–11 (Average 9/11)
Infrastructure & equipment reliability	9	12.3–76.0	4,910	High	9 studies, N = 4,910, CASP 8–11 (Average 9/11)
Privacy & data security issues	5	48.4–80.0	1,593	Moderate	5 studies, N = 1,593, CASP 8–11 (Average 9/11)
Cost & financial barriers	5	31.0–49.0	1,593	Moderate	5 studies, N = 1,593, CASP 8–11 (Average 9/11)
Diagnostic accuracy concerns	6	31.1–63.0	2,699	High	6 studies, N = 2,699, CASP 8–11 (Average 9/11)
Preference for face-to-face	4	48.2–59.0	1,279	Moderate	4 studies, N = 1,279, CASP 8–11 (Average 9/11)

#### Efficiency and cost-effectiveness.

Efficiency and cost-effectiveness were consistently reported as potential facilitators in the included studies. Aboalshamat [19] found that participants viewed teledentistry as a convenient form of care (62.4%), and 56.1% were already using smartphones for consultations, with females more likely to report higher use (p < 0.05). Perceptions of time and cost saving were widespread, ranging from 50% to 70% across four studies [21,22,25,26]. Reducing waiting lists was also highlighted as a potential advantage, with estimates ranging from 65% to 74.1% in three studies [21,23,25].

Perceived benefits to patients can further facilitate acceptance, such as saving money (58.1% to 60% in two studies) [21,25], reducing unnecessary travel (67% to 83% in two studies) [23,25], and enabling remote monitoring (63.5% to 77.7% in three studies) [22,23,25].

During the COVID-19 pandemic, teledentistry was evaluated by practitioners as an effective tool to reduce patient flow (77%) [24], was perceived to ensure safe practice in emergencies (75%) [25], and supported multiple applications (64.6%) [20]. These reported advantages were supported by the high availability of devices (90.5%) and widespread use of electronic medical records (EMR) (72.4%) [27]. Finally, two studies applying the Unified Theory of Acceptance and Use of Technology (UTAUT) model reported high performance expectancy (PE) and effort expectancy (EE), suggesting that teledentistry was perceived as both effective and easy to use [28,29].

#### Professional communication and referrals.

Participants in several studies perceived that teledentistry had the potential to improve professional communication and referral pathways. Perceptions of improved peer communication were reported by 55% to 75% in three studies [21–23,25]. Likewise, 60% to 70% of participants in three studies indicated that teledentistry could potentially enhance communication with patients [21,23,25]. Perceptions of referral enhancement, improved guidance, better advice efficiency, and the potential to shorten waiting lists were also noted, with 55% to 79.5% of participants from four studies expressing agreement [21–23,25].

#### Educational benefits.

Several studies reported that participants appreciated the potential educational role of teledentistry, including its use in continuous education and professional training across five studies (57.1% to 72%) [19–21,23,25]. Patient education, particularly promoting oral health awareness and training in oral hygiene tools, was also perceived as beneficial, with agreement ranging from 54% to 81.4% in three studies [22,24,26]. In addition, one study reported that 57.1% of the participants agreed that teledentistry should be incorporated into dental curricula, highlighting its potential role in academic education [20].

#### Positive perception of technology.

Several studies highlighted participants’ willingness to adopt teledentistry in the future. Aboalshamat [19] reported that 76.8% of participants expressed willingness to practice teledentistry, and 70.7% were willing to support a national initiative to use it, with females being significantly more likely to agree (p < 0.05). Similarly, Alawwad et al. [26] found that 54% were willing to use it in the future and 41% viewed it as a pre-phase to robotics in dental care. Insurance coverage was also seen as a potential enabler, with 60% of participants in two studies supporting its inclusion in insurance plans [21,22]. Nassani et al. [24] further reported that participants showed interest in applying teledentistry (66.3%), acknowledged its potential important future role (66%), and emphasised the need for government initiatives (67.6%).

Other potential facilitators were linked to professional confidence and familiarity with technology. Almazrooa et al. [27] found that 83.8% of participants felt confident that teledentistry had the potential to improve dental practice, with additional perceived benefits in diagnosis (68.9%). Khan et al. [20] also reported familiarity with the concept (63.4%), ease of intraoral camera use (61.4%), and perceived benefits during the pandemic (59.3%), while 49.7% believed it had the potential to enhance dental care. High social influence was also highlighted as a significant predictor of behavioural intention to adopt in the UTAUT model [29].

### Barriers

#### Limited awareness, training, and acceptance.

Limited awareness and knowledge are major reported barriers to the potential implementation of teledentistry. Several studies reported low familiarity among participants, ranging from 17.2% to 38% across five studies [19,20,24,26,27] with Nassani et al. [24] reporting that specialists had significantly greater knowledge (p = 0.001). Alawwad et al. [26] found that 46% did not plan to utilise it in the future, and 65% viewed it as limited in usefulness. Similarly, Nassani et al. [24] reported that only 28.7% believed it could be applied across all branches of dentistry, with just 23.2% reporting currently practising it. The UTAUT model showed low behavioural intention (BI) to use teledentistry, reflecting these issues [29].

Training was another reported challenge, with 75.3% identifying technical difficulties that required further training [20], and 41.4% expressing concern about the time needed to acquire sufficient knowledge [19]. Nassani et al. [24] further highlighted that only 20.2% reported that they had attended lectures on teledentistry, while 79.8% reported a lack of previous training, with specialists and experienced dentists having significantly more exposure (p = 0.001). Participants also perceived teledentistry as having limited value for population education, with agreement ranging from 29% to 47.77% in two studies [19,26]. Dentists’ reluctance added another reported barrier, with 35% thinking such challenges could hinder potential utilisation and 48.7% considering patient compliance to require physical presence [19]. Additionally, 23% disagreed that teledentistry could save time [26]. The UTAUT model further showed low social influence (SI) among students, reflecting the poor knowledge and reduced willingness to practise [28].

#### Infrastructure and equipment reliability.

Infrastructure was consistently reported as a major barrier to the potential implementation of teledentistry. Aboalshamat [19] found that 34.4% of participants perceived a lack of infrastructure, and 42% believed that potential implementation could impose high costs, while Khan et al. [20] reported that 12.3% considered it costly. Another identified issue is equipment compatibility, highlighted by 24.7% to 76% of participants across four studies [20,21,23,25]. Additionally, equipment reliability represented a major concern in four studies ranging from 55.3% to 75% [21–23,25], with Khan et al. [20] further specifying poor internet connection (37.5%) and hardware shortages (25.5%). Scepticism was also evident, as Almazrooa et al. [27] reported that 35.8% disagreed that teledentistry could reduce appointment time, and 29.7% disagreed that it had the potential to improve record quality. In addition, Khan et al. [20] noted that 20.8% were concerned about legal issues, and 23.2% felt it had limited use during the pandemic. Finally, the UTAUT model in two studies indicated low facilitating conditions (FC), reflecting perceived deficiencies in technical infrastructure and lack of preparedness [28,29].

#### Privacy and security concerns.

Privacy and security concerns were consistently reported as major barriers to the potential use of teledentistry. Five studies reported high levels of concern regarding patient confidentiality and privacy, ranging from 48.4% to 80% [19,21–23,25]. In Alshammari and Almaktoom [22], gender showed a statistically significant association with confidentiality concerns (p = 0.008), although the study did not specify which gender expressed greater concerns. Similarly, Alsharif and Al-harbi [23] reported significantly higher privacy concerns among females (p = 0.001).

In addition, obtaining patient consent was highlighted as a perceived challenge, with 56.2% of the participants in Aldhamen and Al Dosari [25], and 70% in Al-Khalifa and AlSheikh [21] reporting it as a concern. Another critical issue was the perceived risk of digital forgery, which was noted by 51.5% to 77% of participants across three studies [21,23,25], with Alsharif and Al-harbi [23] showing that females expressed significantly higher concerns regarding forgery (p = 0.001).

#### Cost and financial barriers.

The financial cost associated with establishing teledentistry services was frequently reported as an anticipated barrier. Between 31% and 44.7% of participants in three studies perceived high set-up costs as an obstacle [21,22,25]. Concerns were also raised regarding patient fees, with 37.3% of participants in Aboalshamat [19] identifying this as a potential barrier. Moreover, 49% of respondents in Alsharif and Al-harbi [23] disagreed that teledentistry had the potential to reduce costs, further highlighting the financial uncertainty surrounding its potential implementation.

#### Diagnostic accuracy concerns.

Concerns regarding diagnostic accuracy were consistently reported as a barrier to the potential adoption of teledentistry. Between 40% and 63% of participants across five studies expressed doubts about the reliability of remote diagnosis [20–23,27]. Specific concerns included the adequacy of diagnostic information to start treatment, reported by 31.1% [27] and 50% [22]. Similarly, 31.3% of participants in Nassani et al. [24] questioned the efficiency of remote examinations, while 50.9% in Khan et al. [20] raised concerns about the ability to provide an accurate diagnosis.

#### Communication method preference.

Preference for traditional communication methods was reported as an anticipated barrier to the potential adoption of teledentistry. Two studies reported that the majority of participants expressed a preference for face-to-face interactions, with limited acceptance of video conferencing as an alternative [21,23]. Another reported issue was the perceived need for an additional appointment to capture photographs for diagnosis, which was reported in four studies and ranged from 48.2% to 59% [21–23,25]. Alsharif and Al-harbi [23] further showed that more females (35%) than males (13%) expressed this potential need, with statistical significance (p = 0.001).

## Discussion

This systematic review synthesised evidence from 11 cross-sectional studies that explored the factors that could influence teledentistry’s potential adoption among dental care providers in Saudi Arabia. Heterogeneity was observed across studies, as some focused on dental students, while others examined practising dentists, and some included both groups. This may explain the variation in the reported perceived barriers and facilitators. Additionally, differences in institutional contexts, infrastructure, and prior exposure to teledentistry likely contributed to inconsistencies in findings. This heterogeneity justifies the choice of narrative synthesis using the SWiM framework rather than conducting a meta-analysis.

Overall, the findings indicate a generally positive perception of teledentistry, as many participants acknowledged its potential to improve access, reduce costs, enhance education, and support training. Several studies highlighted that teledentistry was perceived as an efficient and time-saving tool, particularly to potentially facilitate remote consultations and improve communication with both patients and peers, as well as its reported utility in facilitating access during the COVID-19 pandemic, when maintaining safe and accessible care became a priority. Similar findings were observed globally, where teledentistry adoption was viewed as having a positive influence on dental practices [30–33]. This also explains the high performance expectancy and effort expectancy reported by the two studies that employed the UTAUT model [28,29].

However, perceptions surrounding potential adoption patterns in this review showed that dental care providers were often skeptical of its usefulness, highlighting the limited applicability across all branches of dentistry. Similarly, Ozveren et al. [34] observed that dental care providers were often selective in their willingness to implement it, viewing it as useful only for specific tasks such as follow-ups or triaging cases. This aligns with the wider literature, where surgically oriented specialties tend to perceive teledentistry as having limited applicability due to the need for in-person examination and procedural interventions [30].

Although this review did not analyse students and practising dentists separately, some differences appeared. Students were often characterised by limited knowledge and low social influence [28], while specialists and experienced dentists, despite greater exposure and training, expressed skepticism about diagnostic accuracy [24,27]. This aligns with literature identifying experienced dentists as having less confidence [35]. These findings suggest that greater experience and training may lead to increased awareness of teledentistry’s potential limitations, rather than improving confidence. Similarly, Ghai [36] emphasised that while adequate training and education are essential for increasing acceptance, they are not sufficient on their own; successful integration of teledentistry also requires addressing dental care providers’ concerns regarding diagnostic reliability, technical complexity, and costs, alongside embedding teledentistry into dental curricula.

Limited awareness and insufficient training were consistently reported as major potential barriers in this review. This mirrors the findings of Saberi et al. [31], where many dental care providers initially lacked knowledge of teledentistry, but developed a positive perception and recognised its potential once it was explained to them. A possible explanation for these gaps is the limited integration of teledentistry and telehealth applications into undergraduate curricula and continuous education programmes, which may slow the potential adoption of new technologies in clinical practice [37]. To overcome these challenges, previous research has recommended embedding structured teledentistry training into dental education, increasing hands-on experience, and offering continuous professional development opportunities [38,39].

In addition to educational gaps, participants highlighted technical challenges, including difficulties in using digital platforms, equipment incompatibility, and unstable internet connectivity, all of which are perceived to restrict the smooth future implementation of teledentistry. However, findings from Jarwan et al. [40] challenge this assumption by showing that platforms and infrastructure are widely available in Saudi Arabia, yet reported adoption rates remain low among both practitioners and patients. This suggests that perceived barriers arise less from technical availability and more from motivational and behavioural gaps; despite high performance expectancy and effort expectancy, behavioural intention remained low [29]. This gap highlights the influence of systemic barriers that hinder widespread future adoption rather than technical readiness alone [41].

Interestingly, the UTAUT model revealed a discrepancy in social influence between practising providers and students. While Joseph et al. [29] reported high SI, suggesting peer and institutional support encouraged the intention to adopt, Sharka et al. [28] found low SI among students, likely reflecting limited curricular integration and limited endorsement from universities. This contrast suggests that reported barriers may result from awareness, preparedness, and confidence rather than technological limitations [42]. These findings indicate that the successful future implementation and adoption of teledentistry may largely depend on improving dental care providers’ capabilities and knowledge through targeted educational initiatives, integration into dental curricula, and investment in robust technological infrastructure [43]. These findings explain the low facilitating conditions perceived by practitioners in both Sharka et al. [28] and Joseph et al. [29] in the UTAUT model.

Privacy, data security, and confidentiality emerged as major potential barriers to teledentistry adoption. Findings from Alsharif and Al-harbi [23] and Alshammari and Almaktoom [22] revealed that female practitioners reported significantly higher concerns regarding both privacy and digital forgery, suggesting that they are generally more cautious about patient data handling in remote care. This aligns with Alodhayani et al. [44], who attributed this increased caution among females not only to concerns about handling sensitive patient information but also to cultural and religious considerations, such as showing the face on camera and interacting visually with patients. Alanezi [45] stated that demographic and cultural factors, including gender norms, can affect the potential adoption and acceptance of e-health. These findings highlight that trust plays a major role in practitioners’ potential acceptance of teledentistry, especially when sensitive personal and visual data are involved. Since these concerns are inherently linked to the use of internet-based solutions, clear communication with patients and transparent policies are essential to build confidence and trust in digital platforms [46]. Addressing these issues requires the implementation of standardised protocols, strong protective measures, and institutional policies to promote safe use for both patients and practitioners [47,48].

Financial concerns were frequently reported, particularly regarding the expected high cost of establishing teledentistry services, uncertainty about insurance coverage, and potential patient fees as limiting factors for implementation. The same findings were reported in the literature, where financial cost was seen as a potential barrier to adoption [33,49]. However, both this review and previous research revealed variability across studies, with dental care providers perceiving teledentistry to be cost-effective when supported by appropriate infrastructure and policy frameworks. Estai et al. [50] further highlighted that cost-effectiveness can be enhanced when savings from teledentistry are reinvested in improving infrastructure, creating a more sustainable model of care. These findings indicate that the financial feasibility of teledentistry is highly context-dependent and closely linked to systemic support, suggesting the need for comprehensive long-term cost-effectiveness analyses to establish its value and inform policy decisions [50].

Concerns regarding diagnostic accuracy were consistently observed in this review. Similarly, McLeod et al. [51] identified diagnostic accuracy as one of the main obstacles to the successful implementation of teledentistry. Additionally, Mohamed et al. [52] noted that diagnostic variability can depend on the location of the decayed tooth and the degree of the disease, suggesting that teledentistry’s value may differ depending on clinical context. To address these challenges, Niknam et al. [53] suggested developing standardised protocols for mobile photography to enhance image quality and support more accurate decision-making. However, implementing such protocols may create equity issues, as clinics in low-resource settings may lack the equipment needed to achieve the required image quality, which could further widen disparities in access to care [54]. This highlights that, while technological innovations have the potential to improve diagnostic reliability, there is a need for policies to facilitate equitable access, such as subsidising equipment or offering institutional support for resource-limited clinics [55].

Although this review highlights a generally positive perception of teledentistry among dental care providers, their reported readiness for implementation is still limited due to persistent educational, technical, financial, and ethical barriers. This gap between perceived benefits and actual adoption highlights the potential need for comprehensive institutional and policy strategies, including structured training, continuing education, and improved infrastructure [42]. Additionally, establishing clear guidelines on privacy and data security could further facilitate adoption, aligning with the newly established Personal Data Protection Law (PDPL) in Saudi Arabia [56]. Furthermore, insurance coverage could play an important role in addressing professional and patient concerns [48]. Despite the high performance expectancy and effort expectancy, Joseph et al. [29] found low behavioural intention, indicating that practitioners remain hesitant to incorporate teledentistry into their practice without strong institutional support, clear reimbursement models, and well-defined implementation guidelines. These recommendations represent logical implications derived from the perceived barriers, highlighting key target areas for institutional planning and future implementation research.

Future research should move beyond cross-sectional surveys and focus on interventional and longitudinal studies to evaluate the real-world effectiveness of teledentistry, its impact on patient outcomes, and its role in improving access to dental care across diverse settings [57].

## Limitations

This review has several limitations despite the extensive efforts to identify relevant studies. The evidence is limited to cross-sectional self-reported studies that were conducted in Saudi Arabia, which may restrict generalisability and prevent drawing causal inferences. The reliance on self-reported data in all the studies may have introduced response and recall biases, potentially overestimating positive attitudes or underestimating the barriers to adoption.

Furthermore, heterogeneity in tools and outcomes limited opportunities for statistical comparison and meta-analysis. Additionally, the reliance on vote-counting methodology represents a key limitation, as it does not incorporate study weighting, precision, or effect sizes, preventing quantitative conclusions regarding the precise magnitude of the identified facilitators and barriers. Moreover, although infrastructure readiness was discussed in some studies, no objective assessment of system reliability, cost-effectiveness, or integration into existing dental workflows was conducted, leaving gaps in understanding the practical feasibility of routine implementation. Finally, the limited timeframe, the researcher’s level of experience, and the single author review may have introduced potential selection bias and misinterpretation of some findings.

Future research should focus on robust study designs, including longitudinal and randomised controlled trials, to evaluate the impact of teledentistry on clinical outcomes, patient satisfaction, and cost-effectiveness. Establishing standardised reporting frameworks and incorporating mixed-methods approaches could enhance comparability across studies and improve the synthesis of evidence. Despite these limitations, the findings of this review provide important insights into the opportunities and challenges surrounding teledentistry adoption in Saudi Arabia and highlight critical areas for future research and policy development.

## Conclusion

This systematic review synthesised evidence from cross-sectional studies exploring the potential facilitators and the perceived barriers to the adoption of teledentistry among dental care providers in Saudi Arabia. The findings demonstrate that several factors are perceived to influence the successful future integration of teledentistry into practice. Improved access to care, cost and time efficiency, enhanced professional communication, educational benefits, and a positive perception of technology are acting as strong potential enablers to adoption. On the other hand, persistent, reported barriers, including limited training and awareness, infrastructural issues, privacy and security concerns, diagnostic reliability issues, and preferences for traditional communication methods, are still possibly hindering the widespread implementation of teledentistry into routine dental practices.

These findings reflect key professional perceptions that highlight critical operational and educational gaps rather than definitive implementation outcomes. Consequently, recommendations regarding regulatory frameworks, structured curricular integration, and data protection policies should be interpreted as potential strategies derived from perceived challenges rather than directly proven solutions. Addressing these provider-reported concerns through targeted educational initiatives, technical support, and transparent policy guidelines could foster readiness and facilitate equitable, evidence-based digital health integration aligned with Saudi Vision 2030.

## Acknowledgment

We would like to express our sincere gratitude to Ms Judy Jenkins, Senior Lecturer and Programme Director for the MSc in Health Informatics at Swansea University, for her constructive review, valuable feedback, and guidance on an earlier version of this manuscript.

## Supporting information

S1 Checklist PRISMA 2020 checklist. (PDF)

S1 Table Search terms and results for each database. (XLSX)

S2 Table PICO specifications. (XLSX)

S3 Table Inclusion and exclusion criteria. (XLSX)

S4 Table Data extraction table. (XLSX)

S5 Table Facilitators grouped into themes. (XLSX)

S6 Table Barriers grouped into themes. (XLSX)
