# Supplementary material for: The perceived facilitators and barriers to the potential adoption of teledentistry among dental care providers in Saudi Arabia: A systematic review
Source: PLoS One. 2026 Sep 28;21(9):e0359436. doi: 10.1371/journal.pone.0359436 (PMC13618885; doi:10.1371/journal.pone.0359436)
Supplement: Supplementary file 2 [file pone.0359436.s009.docx]

**Dear editor and reviewers,**

Thank you for your constructive and thorough review of our manuscript. We sincerely appreciate the time and effort invested to strengthen the quality, methodology, and clarity of our manuscript. We have carefully addressed all major and minor comments. Below is a point-by-point response of all the revisions made in the revised manuscript.

**Response to editor**

**Comment:** We note that there is identifying data in the Supporting Information file <S1 Table >. Due to the inclusion of these potentially identifying data, we have removed this file from your file inventory. Prior to sharing human research participant data, authors should consult with an ethics committee to ensure data are shared in accordance with participant consent and all applicable local laws.

**Response:** Thank you for bringing this to our attention. We would like to clarify that S1 Table contains exclusively database search syntaxes, MeSH terms, and search query strings. It contains no human subject data, individual clinical records, or personal identifiable information. We have re-uploaded S1 Table in the file inventory accordingly.

**Response to reviewer #1**

**Comment 1**: Author Summary: Is this section really necessary?

**Response**: Thank you for bringing this to our attention. We agree with the reviewer. As per PLOS ONE manuscript guidelines, an “Author Summary” section is not required. Consequently, we have removed it from the revised manuscript.

**Comment 2**: The conclusion is too long. I recommend reducing it to a maximum of two paragraphs.

**Response**: We fully agree with the reviewer’s recommendation. We have restructured the conclusion section into two focused paragraphs that summarise the core synthesis findings and frame the policy and educational implications appropriately.

**Comment 3**: I understand that this systematic review aims to raise awareness of teledentistry in Saudi Arabia. However, I believe it would be important to highlight its relevance beyond Saudi Arabia and to discuss how teledentistry has been successfully implemented and used in other countries around the world.

**Response**: Thank you. The global relevance of our findings is already contextualised within the discussion. Specifically, we compared the findings in Saudi Arabia with wider international literature, noting that similar perceived facilitators and barriers are observed globally.

**Response to reviewer #2**

**Major comments**

**Comment 1: Interpretation of "adoption" versus perceptions:**
The included studies are almost exclusively cross-sectional questionnaire surveys assessing awareness, knowledge, attitudes, willingness, and perceptions. These outcomes do not directly measure implementation or adoption of teledentistry. Throughout the manuscript, the terms "adoption" and "implementation" are frequently used interchangeably with perceived facilitators and barriers. The authors should consistently clarify that the review synthesizes perceived determinants rather than actual adoption or implementation outcomes.

**Response**: Thank you for your valuable comments. We completely agree with this fundamental point. We have revised the manuscript to ensure consistent terminology, replacing claims of actual implementation with terms such as “perceived facilitators”, “reported barriers”, and “potential adoption”.

**Comment 2: Evidence certainty assessment:**
The manuscript states that the approach aligns with the principles of the GRADE framework. However, the presented evidence classification is a novel scoring system based on CASP score, number of studies, and cumulative sample size rather than a formal GRADE assessment. This should either be presented explicitly as an author-developed approach without reference to GRADE or replaced by an established certainty-of-evidence methodology. As currently written, the description may mislead readers into assuming that formal GRADE methodology was applied.

**Response**: Thank you for this important methodological clarification. We have updated the “**Data synthesis**” to present our certainty assessment strictly as an author-developed framework. All references to the GRADE framework have been removed from the manuscript to avoid misleading.

**Comment 3: Search strategy:**
The manuscript should provide greater justification for the search strategy. Based on the methods, the search appears primarily centered on the concepts "Teledentistry" and "Saudi Arabia." The authors should demonstrate that the search strategy was sufficiently comprehensive to identify all potentially relevant studies and discuss whether additional terminology related to telehealth, telemedicine, remote consultation, oral health services, and other synonymous concepts was considered.

**Response:** We appreciate this suggestion. We have revised the “Search Strategy” section to clarify that “Teledentistry” is a recognised MeSH term specific to remote oral health services. Broader terms like “Telehealth” were excluded to maintain specificity and avoid non-dental literature, while relevant synonyms (Teledental, dental telemedicne) were incorporated, all of this is now reflected in the manuscript. Full search terms are provided in S1 Table.

**Comment 4: Quality appraisal:**
The authors used the CASP checklist for cross-sectional studies. Although acceptable, this choice should be justified, particularly because alternative validated instruments (such as the JBI or AXIS tools) are commonly used for cross-sectional studies. A brief rationale would strengthen the methodological transparency.

**Response:** Thank you. We have added a breif rationale in the “Quality appraisal” section explaining that the CASP checklist was selected because it provide a structured and transparent framework tailored to evaluate key survey methodology parameters. Including recruitment strategies, measurement bias, statistical rigor, and applicability.

**Comment 5: Vote-counting methodology:**
The use of SWiM and narrative synthesis is appropriate given the heterogeneity of the included studies. However, readers should be reminded more explicitly that vote counting does not account for study precision, weighting, or effect size, and therefore the reported themes should not be interpreted as quantitative evidence of importance or magnitude.

**Response:** We agree with this comment. We have explicitly updated both the “Data synthesis” and “Limitations” sections to state that vote-counting does not account for study precision, weighting, or effect sizes, and that reported frequencies reflect qualitative thematic categories rather than quantitative effect magnitudes.

**Comment 6: Strength of conclusions:**
Several statements within the Discussion and Conclusion extend beyond the available evidence. Recommendations regarding regulatory frameworks, reimbursement models, educational reforms, and implementation strategies are reasonable but should be presented as implications derived from perceived barriers rather than conclusions directly supported by the included cross-sectional surveys.

**Response:** We fully agree. We have carefully revised the “Discussion” and “Conclusion” sections to tone down our recommendations. Policy, curricular, and regulatory recommendations are now explicitly presented as logical implications derived from perceived challenges rather than direct empirical conclusions.

**Minor Comments**

**Comment 1:** The manuscript would benefit from consistently using either "dental care providers" or "dentists and dental students" throughout to improve terminology consistency.

**Response:** Thank you. We have standardised the manuscript terminology to consistantly use “dental care providers” throughout the text, specifiying subgroups only where explicit comparative findings are discussed.

**Comment 2:** The Discussion could more clearly distinguish findings relating to dental students from those relating to practicing dentists where possible.

**Response:** The discussion section explicitly differentiates findings relating to dental students (e.g., lower baseline knowledge and social influence) from those of practising dentists and specialists (e.g., elevated concerns regarding diagnostic accuracy and privacy) where possible.

**Comment 3:**  Please proofread the manuscript for minor typographical and grammatical errors (for example, "literature" is misspelled in the Ethics section).

**Response:** Thank you for pointing this out. The error has been corrected, and a thorough proofreading was conducted across the manuscript.

**Comment 4:** Consider reporting the principal limitations of vote counting more explicitly within the limitations section.

**Response:** We have updated the “Limitations” section to explicitly detail the limitations of vote-counting methodology regarding precesion, study weighting, and effect sizes.

**Comment 5:** Some percentage ranges are presented without sufficient contextual explanation. Where feasible, indicate the number of contributing studies alongside the reported ranges to improve interpretability.

**Response:** We have updated the “Results” section to include the exact numbers of contributing studies alongside every reported percentage range across all facilitator and barrier themes.

We hope that these revisions satisfactorily address all comments and that the manuscript is now suitable for publication in PLOS ONE.

Sincerely,

Amjad Hakami & Sarika Mohan

Department of Health Data Science, Swansea University
