# Supplementary material for: The perceived facilitators and barriers to the potential adoption of teledentistry among dental care providers in Saudi Arabia: A systematic review
Source: PLoS One. 2026 Sep 28;21(9):e0359436. doi: 10.1371/journal.pone.0359436 (PMC13618885; doi:10.1371/journal.pone.0359436)
Supplement: Supplementary file 3 [file pone.0359436.s010.docx]

**Dear Editor and Reviewer,**

Thank you for your constructive and thorough review of our manuscript. We sincerely appreciate the time and effort invested to strengthen the quality, methodology, and clarity of our manuscript. We have carefully addressed all three comments. Below is a point-by-point response of all the revisions made in the revised manuscript.

**Response to reviewer #2**

**Comment:** Table 2 is still titled “Strength of evidence based on SWiM guidelines,” whereas the Methods now correctly describe the high/moderate/low classification as an author-developed framework. The table title should therefore be revised so that the classification is not attributed to SWiM.

**Response:** Thank you for bringing this to our attention. We have changed Table 2 title to *“Strength of evidence based on themes”.*

**Comment:** The authors should also clarify how the CASP criterion was applied when assigning evidence strength, particularly as some themes classified as “High” include studies with CASP scores below 9 and some “Moderate” themes have an average CASP score of 9.

**Response**: Thank you for this important methodological clarification. We have updated the "Data synthesis" section to clearly define how the CASP criterion and evidence strength thresholds were applied. Specifically, the framework evaluates the average CASP score alongside study volume and cumulative sample size. A theme was rated as High if it met all three criteria simultaneously. A theme was classified as Moderate if it failed to meet the sample size or study count thresholds required for High, even if its average CASP score was ≥ 9. This clarifies why themes with an average CASP score of 9 were classified as Moderate and confirms that the threshold for High reflects the thematic average quality rather than requiring every individual study to score 9 or above.

**Comment**: a few statements remain more definitive than warranted by the underlying cross-sectional perception data. For example, the Abstract states that the proposed measures “can ensure equitable adoption.” This should be expressed more cautiously (e.g., that such measures may facilitate or support equitable adoption). Similar definitive wording should be checked throughout the manuscript.

**Response**: We agree with the reviewer's observation. We have carefully revised the manuscript to soften definitive phrasing and ensure cautious, non-causal language throughout that accurately reflects cross-sectional perception data. These changes were reflected across the Abstract, Introduction, and Discussion sections.

We hope that these revisions satisfactorily address all comments and that the manuscript is now suitable for publication in PLOS ONE.

Sincerely,

Amjad Hakami & Sarika Mohan

Department of Health Data Science, Swansea University
