# Supplementary material for: The perceived facilitators and barriers to the potential adoption of teledentistry among dental care providers in Saudi Arabia: A systematic review
Source: PLoS One. 2026 Sep 28;21(9):e0359436. doi: 10.1371/journal.pone.0359436 (PMC13618885; doi:10.1371/journal.pone.0359436)
Supplement: Peer review history

# Author comment: Author response to Decision Letter 0

**DOI:** 10.1371/journal.pone.0359436.r001

**Transfer Alert**

This paper was transferred from another journal. As a result, its full editorial history (including decision letters, peer reviews and author responses) may not be present.

23 Apr 2026

# Peer review: Decision Letter 0

**DOI:** 10.1371/journal.pone.0359436.r002

**Authors:** Muhammad Umer

16 Jul 2026

Dear Dr. Hakami,

Thank you for submitting your manuscript to PLOS ONE. After careful consideration, we feel that it has merit but does not fully meet PLOS ONE’s publication criteria as it currently stands. Therefore, we invite you to submit a revised version of the manuscript that addresses the points raised during the review process.

===========================

Reviewer 2 has identified two substantive methodological concerns that materially affect the interpretation of the review's findings and conclusions. First, the manuscript interprets data on participants' perceptions and willingness as evidence of actual *adoption* , which represents an overstatement of the available evidence and requires more accurate terminology throughout the manuscript. Second, the manuscript describes a study quality assessment approach as being aligned with the GRADE framework, despite employing a bespoke scoring system that does not conform to established GRADE methodology. This issue should be corrected to ensure methodological accuracy and to avoid misleading readers regarding the certainty of the evidence. These concerns directly influence the strength and interpretation of the review's conclusions and should therefore be considered essential revisions prior to acceptance. In contrast, the requests for additional justification of the search strategy and the rationale for selecting the CASP appraisal tool are primarily matters of methodological transparency and reporting. Although these points should be adequately addressed to improve the manuscript's clarity and reproducibility, they do not fundamentally compromise the validity of the review.

==============================

Please submit your revised manuscript by Aug 30 2026 11:59PM. If you will need more time than this to complete your revisions, please reply to this message or contact the journal office at plosone@plos.org. When you're ready to submit your revision, log on to https://www.editorialmanager.com/pone/ and select the 'Submissions Needing Revision' folder to locate your manuscript file.

- A letter that responds to each point raised by the academic editor and reviewer(s). You should upload this letter as a separate file labeled 'Response to Reviewers'.
- A marked-up copy of your manuscript that highlights changes made to the original version. You should upload this as a separate file labeled 'Revised Manuscript with Track Changes'.
- An unmarked version of your revised paper without tracked changes. You should upload this as a separate file labeled 'Manuscript'.

If you would like to make changes to your financial disclosure, please include your updated statement in your cover letter. Guidelines for resubmitting your figure files are available below the reviewer comments at the end of this letter.

If applicable, we recommend that you deposit your laboratory protocols in protocols.io to enhance the reproducibility of your results. Protocols.io assigns your protocol its own identifier (DOI) so that it can be cited independently in the future. For instructions see: https://journals.plos.org/plosone/s/submission-guidelines#loc-laboratory-protocols. Additionally, PLOS ONE offers an option for publishing peer-reviewed Lab Protocol articles, which describe protocols hosted on protocols.io. Read more information on sharing protocols at https://plos.org/protocols?utm_medium=editorial-email&utm_source=authorletters&utm_campaign=protocols.

As the corresponding author, your ORCID iD is verified in the submission system and will appear in the published article. PLOS supports the use of ORCID, and we encourage all coauthors to register for an ORCID iD and use it as well. Please encourage your coauthors to verify their ORCID iD within the submission system before final acceptance, as unverified ORCID iDs will not appear in the published article. *Only*  the individual author can complete the verification step; PLOS staff *cannot*  verify ORCID iDs on behalf of authors.

We look forward to receiving your revised manuscript.

Kind regards,

Muhammad Farooq Umer, PhD Epidemiology and Health Statistics

Academic Editor

PLOS One

Journal Requirements:

When submitting your revision, we need you to address these additional requirements.

1. Please ensure that your manuscript meets PLOS ONE's style requirements, including those for file naming. The PLOS ONE style templates can be found at

https://journals.plos.org/plosone/s/file?id=wjVg/PLOSOne_formatting_sample_main_body.pdf and

https://journals.

2. We note that there is identifying data in the Supporting Information file <S1 Table >. Due to the inclusion of these potentially identifying data, we have removed this file from your file inventory. Prior to sharing human research participant data, authors should consult with an ethics committee to ensure data are shared in accordance with participant consent and all applicable local laws.

Data sharing should never compromise participant privacy. It is therefore not appropriate to publicly share personally identifiable data on human research participants. The following are examples of data that should not be shared:

-Name, initials, physical address

-Ages more specific than whole numbers

-Internet protocol (IP) address

-Specific dates (birth dates, death dates, examination dates, etc.)

-Contact information such as phone number or email address

-Location data

-ID numbers that seem specific (long numbers, include initials, titled “Hospital ID”) rather than random (small numbers in numerical order)

Data that are not directly identifying may also be inappropriate to share, as in combination they can become identifying. For example, data collected from a small group of participants, vulnerable populations, or private groups should not be shared if they involve indirect identifiers (such as sex, ethnicity, location, etc.) that may risk the identification of study participants.

Additional guidance on preparing raw data for publication can be found in our Data Policy (https://journals.plos.org/plosone/s/data-availability#loc-human-research-participant-data-and-other-sensitive-data) and in the following article: http://www.bmj.com/content/340/bmj.c181.long.

Please remove or anonymize all personal information (<specific identifying information in file to be removed>), ensure that the data shared are in accordance with participant consent, and re-upload a fully anonymized data set. Please note that spreadsheet columns with personal information must be removed and not hidden as all hidden columns will appear in the published file.

3. If the reviewer comments include a recommendation to cite specific previously published works, please review and evaluate these publications to determine whether they are relevant and should be cited. There is no requirement to cite these works unless the editor has indicated otherwise.

[Note: HTML markup is below. Please do not edit.]

Reviewers' comments:

Reviewer's Responses to Questions

**Comments to the Author**

1. Is the manuscript technically sound, and do the data support the conclusions?

Reviewer #1: Yes

Reviewer #2: Yes

**********

2. Has the statistical analysis been performed appropriately and rigorously?

Reviewer #1: Yes

Reviewer #2: Yes

**********

3. Have the authors made all data underlying the findings in their manuscript fully available?

The PLOS Data policy

Reviewer #1: Yes

Reviewer #2: Yes

**********

4. Is the manuscript presented in an intelligible fashion and written in standard English?

Reviewer #1: Yes

Reviewer #2: Yes

**********

Reviewer #1: Very interesting article.

Abstract: Well written.

Author Summary: Is this section really necessary?

Introduction: OK.

Methods: Well written and easy to understand.

Results: OK.

Discussion: OK.

Limitations: OK.

Conclusion: The conclusion is too long. I recommend reducing it to a maximum of two paragraphs.

I understand that this systematic review aims to raise awareness of teledentistry in Saudi Arabia. However, I believe it would be important to highlight its relevance beyond Saudi Arabia and to discuss how teledentistry has been successfully implemented and used in other countries around the world.

Reviewer #2: Thank you for the opportunity to review this systematic review addressing facilitators and barriers to teledentistry adoption among dental care providers in Saudi Arabia. The topic is timely, relevant to digital health transformation and Saudi Vision 2030, and the manuscript is generally well organized and clearly written. The review follows a PRISMA-based structure, is prospectively registered in PROSPERO, and provides a useful synthesis of the available literature.

However, I identified several methodological and reporting issues that should be addressed before the manuscript is suitable for publication. My comments are intended to strengthen the scientific rigor and improve the interpretation of the findings.

Major comments

1. Interpretation of "adoption" versus perceptions

The included studies are almost exclusively cross-sectional questionnaire surveys assessing awareness, knowledge, attitudes, willingness, and perceptions. These outcomes do not directly measure implementation or adoption of teledentistry. Throughout the manuscript, the terms "adoption" and "implementation" are frequently used interchangeably with perceived facilitators and barriers. The authors should consistently clarify that the review synthesizes perceived determinants rather than actual adoption or implementation outcomes.

2. Evidence certainty assessment

The manuscript states that the approach aligns with the principles of the GRADE framework. However, the presented evidence classification is a novel scoring system based on CASP score, number of studies, and cumulative sample size rather than a formal GRADE assessment. This should either be presented explicitly as an author-developed approach without reference to GRADE or replaced by an established certainty-of-evidence methodology. As currently written, the description may mislead readers into assuming that formal GRADE methodology was applied.

3. Search strategy

The manuscript should provide greater justification for the search strategy. Based on the methods, the search appears primarily centered on the concepts "Teledentistry" and "Saudi Arabia." The authors should demonstrate that the search strategy was sufficiently comprehensive to identify all potentially relevant studies and discuss whether additional terminology related to telehealth, telemedicine, remote consultation, oral health services, and other synonymous concepts was considered.

4. Quality appraisal

The authors used the CASP checklist for cross-sectional studies. Although acceptable, this choice should be justified, particularly because alternative validated instruments (such as the JBI or AXIS tools) are commonly used for cross-sectional studies. A brief rationale would strengthen the methodological transparency.

5. Vote-counting methodology

The use of SWiM and narrative synthesis is appropriate given the heterogeneity of the included studies. However, readers should be reminded more explicitly that vote counting does not account for study precision, weighting, or effect size, and therefore the reported themes should not be interpreted as quantitative evidence of importance or magnitude.

6. Strength of conclusions

Several statements within the Discussion and Conclusion extend beyond the available evidence. Recommendations regarding regulatory frameworks, reimbursement models, educational reforms, and implementation strategies are reasonable but should be presented as implications derived from perceived barriers rather than conclusions directly supported by the included cross-sectional surveys.

Minor comments

1. The manuscript would benefit from consistently using either "dental care providers" or "dentists and dental students" throughout to improve terminology consistency.

2. The Discussion could more clearly distinguish findings relating to dental students from those relating to practicing dentists where possible.

3. Please proofread the manuscript for minor typographical and grammatical errors (for example, "literature" is misspelled in the Ethics section).

4. Consider reporting the principal limitations of vote counting more explicitly within the limitations section.

5. Some percentage ranges are presented without sufficient contextual explanation. Where feasible, indicate the number of contributing studies alongside the reported ranges to improve interpretability.

Overall, this review addresses an important topic and has the potential to make a useful contribution to the literature. Addressing the methodological concerns outlined above would substantially strengthen the manuscript and improve confidence in the conclusions.

**********

what does this mean?). If published, this will include your full peer review and any attached files.

If you choose “no”, your identity will remain anonymous but your review may still be made public.

**Do you want your identity to be public for this peer review?**  For information about this choice, including consent withdrawal, please see our Privacy Policy

Reviewer #1: No

Reviewer #2: **Yes:**  Muhammad Zubair Ahmad

**********

[NOTE: If reviewer comments were submitted as an attachment file, they will be attached to this email and accessible via the submission site. Please log into your account, locate the manuscript record, and check for the action link "View Attachments". If this link does not appear, there are no attachment files.]

To ensure your figures meet our technical requirements, please review our figure guidelines: https://journals.plos.org/plosone/s/figures

You may also use PLOS’s free figure tool, NAAS, to help you prepare publication quality figures: https://journals.plos.org/plosone/s/figures#loc-tools-for-figure-preparation.

NAAS will assess whether your figures meet our technical requirements by comparing each figure against our figure specifications.

# Author comment: Author response to Decision Letter 1

**DOI:** 10.1371/journal.pone.0359436.r003

11 Aug 2026

Dear editor and reviewers,

Thank you for your constructive and thorough review of our manuscript. We sincerely appreciate the time and effort invested to strengthen the quality, methodology, and clarity of our manuscript. We have carefully addressed all major and minor comments. Below is a point-by-point response of all the revisions made in the revised manuscript.

Response to editor

Comment: We note that there is identifying data in the Supporting Information file <S1 Table >. Due to the inclusion of these potentially identifying data, we have removed this file from your file inventory. Prior to sharing human research participant data, authors should consult with an ethics committee to ensure data are shared in accordance with participant consent and all applicable local laws.

Response: Thank you for bringing this to our attention. We would like to clarify that S1 Table contains exclusively database search syntaxes, MeSH terms, and search query strings. It contains no human subject data, individual clinical records, or personal identifiable information. We have re-uploaded S1 Table in the file inventory accordingly.

Response to reviewer #1

Comment 1: Author Summary: Is this section really necessary?

Response: Thank you for bringing this to our attention. We agree with the reviewer. As per PLOS ONE manuscript guidelines, an “Author Summary” section is not required. Consequently, we have removed it from the revised manuscript.

Comment 2: The conclusion is too long. I recommend reducing it to a maximum of two paragraphs.

Response: We fully agree with the reviewer’s recommendation. We have restructured the conclusion section into two focused paragraphs that summarise the core synthesis findings and frame the policy and educational implications appropriately.

Comment 3: I understand that this systematic review aims to raise awareness of teledentistry in Saudi Arabia. However, I believe it would be important to highlight its relevance beyond Saudi Arabia and to discuss how teledentistry has been successfully implemented and used in other countries around the world.

Response: Thank you. The global relevance of our findings is already contextualised within the discussion. Specifically, we compared the findings in Saudi Arabia with wider international literature, noting that similar perceived facilitators and barriers are observed globally.

Response to reviewer #2

Major comments

Comment 1: Interpretation of "adoption" versus perceptions:

The included studies are almost exclusively cross-sectional questionnaire surveys assessing awareness, knowledge, attitudes, willingness, and perceptions. These outcomes do not directly measure implementation or adoption of teledentistry. Throughout the manuscript, the terms "adoption" and "implementation" are frequently used interchangeably with perceived facilitators and barriers. The authors should consistently clarify that the review synthesizes perceived determinants rather than actual adoption or implementation outcomes.

Response: Thank you for your valuable comments. We completely agree with this fundamental point. We have revised the manuscript to ensure consistent terminology, replacing claims of actual implementation with terms such as “perceived facilitators”, “reported barriers”, and “potential adoption”.

Comment 2: Evidence certainty assessment:

The manuscript states that the approach aligns with the principles of the GRADE framework. However, the presented evidence classification is a novel scoring system based on CASP score, number of studies, and cumulative sample size rather than a formal GRADE assessment. This should either be presented explicitly as an author-developed approach without reference to GRADE or replaced by an established certainty-of-evidence methodology. As currently written, the description may mislead readers into assuming that formal GRADE methodology was applied.

Response: Thank you for this important methodological clarification. We have updated the “Data synthesis” to present our certainty assessment strictly as an author-developed framework. All references to the GRADE framework have been removed from the manuscript to avoid misleading.

Comment 3: Search strategy:

The manuscript should provide greater justification for the search strategy. Based on the methods, the search appears primarily centered on the concepts "Teledentistry" and "Saudi Arabia." The authors should demonstrate that the search strategy was sufficiently comprehensive to identify all potentially relevant studies and discuss whether additional terminology related to telehealth, telemedicine, remote consultation, oral health services, and other synonymous concepts was considered.

Response: We appreciate this suggestion. We have revised the “Search Strategy” section to clarify that “Teledentistry” is a recognised MeSH term specific to remote oral health services. Broader terms like “Telehealth” were excluded to maintain specificity and avoid non-dental literature, while relevant synonyms (Teledental, dental telemedicne) were incorporated, all of this is now reflected in the manuscript. Full search terms are provided in S1 Table.

Comment 4: Quality appraisal:

The authors used the CASP checklist for cross-sectional studies. Although acceptable, this choice should be justified, particularly because alternative validated instruments (such as the JBI or AXIS tools) are commonly used for cross-sectional studies. A brief rationale would strengthen the methodological transparency.

Response: Thank you. We have added a breif rationale in the “Quality appraisal” section explaining that the CASP checklist was selected because it provide a structured and transparent framework tailored to evaluate key survey methodology parameters. Including recruitment strategies, measurement bias, statistical rigor, and applicability.

Comment 5: Vote-counting methodology:

The use of SWiM and narrative synthesis is appropriate given the heterogeneity of the included studies. However, readers should be reminded more explicitly that vote counting does not account for study precision, weighting, or effect size, and therefore the reported themes should not be interpreted as quantitative evidence of importance or magnitude.

Response: We agree with this comment. We have explicitly updated both the “Data synthesis” and “Limitations” sections to state that vote-counting does not account for study precision, weighting, or effect sizes, and that reported frequencies reflect qualitative thematic categories rather than quantitative effect magnitudes.

Comment 6: Strength of conclusions:

Several statements within the Discussion and Conclusion extend beyond the available evidence. Recommendations regarding regulatory frameworks, reimbursement models, educational reforms, and implementation strategies are reasonable but should be presented as implications derived from perceived barriers rather than conclusions directly supported by the included cross-sectional surveys.

Response: We fully agree. We have carefully revised the “Discussion” and “Conclusion” sections to tone down our recommendations. Policy, curricular, and regulatory recommendations are now explicitly presented as logical implications derived from perceived challenges rather than direct empirical conclusions.

Minor Comments

Comment 1: The manuscript would benefit from consistently using either "dental care providers" or "dentists and dental students" throughout to improve terminology consistency.

Response: Thank you. We have standardised the manuscript terminology to consistantly use “dental care providers” throughout the text, specifiying subgroups only where explicit comparative findings are discussed.

Comment 2: The Discussion could more clearly distinguish findings relating to dental students from those relating to practicing dentists where possible.

Response: The discussion section explicitly differentiates findings relating to dental students (e.g., lower baseline knowledge and social influence) from those of practising dentists and specialists (e.g., elevated concerns regarding diagnostic accuracy and privacy) where possible.

Comment 3: Please proofread the manuscript for minor typographical and grammatical errors (for example, "literature" is misspelled in the Ethics section).

Response: Thank you for pointing this out. The error has been corrected, and a thorough proofreading was conducted across the manuscript.

Comment 4: Consider reporting the principal limitations of vote counting more explicitly within the limitations section.

Response: We have updated the “Limitations” section to explicitly detail the limitations of vote-counting methodology regarding precesion, study weighting, and effect sizes.

Comment 5: Some percentage ranges are presented without sufficient contextual explanation. Where feasible, indicate the number of contributing studies alongside the reported ranges to improve interpretability.

Response: We have updated the “Results” section to include the exact numbers of contributing studies alongside every reported percentage range across all facilitator and barrier themes.

We hope that these revisions satisfactorily address all comments and that the manuscript is now suitable for publication in PLOS ONE.

Sincerely,

Amjad Hakami & Sarika Mohan

Department of Health Data Science, Swansea University

Attachment Submitted filename: Response to editor and reviewers.docx

# Peer review: Decision Letter 1

**DOI:** 10.1371/journal.pone.0359436.r004

**Authors:** Muhammad Umer

9 Sep 2026

Dear Dr. Hakami,

Thank you for submitting your manuscript to PLOS One. After careful consideration, we feel that it has merit but does not fully meet PLOS One’s publication criteria as it currently stands. Therefore, we invite you to submit a revised version of the manuscript that addresses the points raised during the review process.

Although the major concerns have been addressed and the Reviewer 2 has clearly acknowledged them, yet, there are a few minor but important concerns remian to be resolved.

Please submit your revised manuscript by Oct 24 2026 11:59PM. If you will need more time than this to complete your revisions, please reply to this message or contact the journal office at plosone@plos.org. When you're ready to submit your revision, log on to https://www.editorialmanager.com/pone/ and select the 'Submissions Needing Revision' folder to locate your manuscript file.

- A letter that responds to each point raised by the academic editor and reviewer(s). You should upload this letter as a separate file labeled 'Response to Reviewers'.
- A marked-up copy of your manuscript that highlights changes made to the original version. You should upload this as a separate file labeled 'Revised Manuscript with Track Changes'.
- An unmarked version of your revised paper without tracked changes. You should upload this as a separate file labeled 'Manuscript'.

If you would like to make changes to your financial disclosure, please include your updated statement in your cover letter. Guidelines for resubmitting your figure files are available below the reviewer comments at the end of this letter.

If applicable, we recommend that you deposit your laboratory protocols in protocols.io to enhance the reproducibility of your results. Protocols.io assigns your protocol its own identifier (DOI) so that it can be cited independently in the future. For instructions see: https://journals.plos.org/plosone/s/submission-guidelines#loc-laboratory-protocols. Additionally, PLOS One offers an option for publishing peer-reviewed Lab Protocol articles, which describe protocols hosted on protocols.io. Read more information on sharing protocols at https://plos.org/protocols?utm_medium=editorial-email&utm_source=authorletters&utm_campaign=protocols.

As the corresponding author, your ORCID iD is verified in the submission system and will appear in the published article. PLOS supports the use of ORCID, and we encourage all coauthors to register for an ORCID iD and use it as well. Please encourage your coauthors to verify their ORCID iD within the submission system before final acceptance, as unverified ORCID iDs will not appear in the published article. *Only*  the individual author can complete the verification step; PLOS staff *cannot*  verify ORCID iDs on behalf of authors.

We look forward to receiving your revised manuscript.

Kind regards,

Muhammad Farooq Umer, PhD Epidemiology and Health Statistics

Academic Editor

PLOS One

Journal Requirements:

If the reviewer comments include a recommendation to cite specific previously published works, please review and evaluate these publications to determine whether they are relevant and should be cited. There is no requirement to cite these works unless the editor has indicated otherwise.

Please review your reference list to ensure that it is complete and correct. If you have cited papers that have been retracted, please include the rationale for doing so in the manuscript text, or remove these references and replace them with relevant current references. Any changes to the reference list should be mentioned in the rebuttal letter that accompanies your revised manuscript. If you need to cite a retracted article, indicate the article’s retracted status in the References list and also include a citation and full reference for the retraction notice.

[Note: HTML markup is below. Please do not edit.]

Reviewers' comments:

Reviewer's Responses to Questions

**Comments to the Author**

Reviewer #2: All comments have been addressed

**********

2. Is the manuscript technically sound, and do the data support the conclusions?

Reviewer #2: Yes

**********

3. Has the statistical analysis been performed appropriately and rigorously?

Reviewer #2: Yes

**********

4. Have the authors made all data underlying the findings in their manuscript fully available?

The PLOS Data policy

Reviewer #2: Yes

**********

5. Is the manuscript presented in an intelligible fashion and written in standard English?

Reviewer #2: Yes

**********

Reviewer #2: Thank you for carefully addressing the comments raised during the previous round. The manuscript has been substantially improved, and my major concerns regarding the distinction between perceived determinants and actual adoption, the inappropriate reference to GRADE, and the limitations of the narrative synthesis have been satisfactorily addressed.

A few minor issues remain before publication. First, Table 2 is still titled “Strength of evidence based on SWiM guidelines,” whereas the Methods now correctly describe the high/moderate/low classification as an author-developed framework. The table title should therefore be revised so that the classification is not attributed to SWiM. The authors should also clarify how the CASP criterion was applied when assigning evidence strength, particularly as some themes classified as “High” include studies with CASP scores below 9 and some “Moderate” themes have an average CASP score of 9.

Finally, a few statements remain more definitive than warranted by the underlying cross-sectional perception data. For example, the Abstract states that the proposed measures “can ensure equitable adoption.” This should be expressed more cautiously (e.g., that such measures may facilitate or support equitable adoption). Similar definitive wording should be checked throughout the manuscript.

These are relatively minor issues, and the substantive concerns raised in the previous review have otherwise been adequately addressed.

**********

what does this mean?). If published, this will include your full peer review and any attached files.

If you choose “no”, your identity will remain anonymous but your review may still be made public.

**Do you want your identity to be public for this peer review?**  For information about this choice, including consent withdrawal, please see our Privacy Policy

Reviewer #2: **Yes:**  Muhammad Zubair Ahmad

**********

[NOTE: If reviewer comments were submitted as an attachment file, they will be attached to this email and accessible via the submission site. Please log into your account, locate the manuscript record, and check for the action link "View Attachments". If this link does not appear, there are no attachment files.]

To ensure your figures meet our technical requirements, please review our figure guidelines: https://journals.plos.org/plosone/s/figures

You may also use PLOS’s free figure tool, NAAS, to help you prepare publication quality figures: https://journals.plos.org/plosone/s/figures#loc-tools-for-figure-preparation.

NAAS will assess whether your figures meet our technical requirements by comparing each figure against our figure specifications.

# Author comment: Author response to Decision Letter 2

**DOI:** 10.1371/journal.pone.0359436.r005

10 Sep 2026

Dear Editor and Reviewer,

Thank you for your constructive and thorough review of our manuscript. We sincerely appreciate the time and effort invested to strengthen the quality, methodology, and clarity of our manuscript. We have carefully addressed all three comments. Below is a point-by-point response of all the revisions made in the revised manuscript.

Response to reviewer #2

Comment: Table 2 is still titled “Strength of evidence based on SWiM guidelines,” whereas the Methods now correctly describe the high/moderate/low classification as an author-developed framework. The table title should therefore be revised so that the classification is not attributed to SWiM.

Response: Thank you for bringing this to our attention. We have changed Table 2 title to “Strength of evidence based on themes”.

Comment: The authors should also clarify how the CASP criterion was applied when assigning evidence strength, particularly as some themes classified as “High” include studies with CASP scores below 9 and some “Moderate” themes have an average CASP score of 9.

Response: Thank you for this important methodological clarification. We have updated the "Data synthesis" section to clearly define how the CASP criterion and evidence strength thresholds were applied. Specifically, the framework evaluates the average CASP score alongside study volume and cumulative sample size. A theme was rated as High if it met all three criteria simultaneously. A theme was classified as Moderate if it failed to meet the sample size or study count thresholds required for High, even if its average CASP score was ≥ 9. This clarifies why themes with an average CASP score of 9 were classified as Moderate and confirms that the threshold for High reflects the thematic average quality rather than requiring every individual study to score 9 or above.

Comment: a few statements remain more definitive than warranted by the underlying cross-sectional perception data. For example, the Abstract states that the proposed measures “can ensure equitable adoption.” This should be expressed more cautiously (e.g., that such measures may facilitate or support equitable adoption). Similar definitive wording should be checked throughout the manuscript.

Response: We agree with the reviewer's observation. We have carefully revised the manuscript to soften definitive phrasing and ensure cautious, non-causal language throughout that accurately reflects cross-sectional perception data. These changes were reflected across the Abstract, Introduction, and Discussion sections.

We hope that these revisions satisfactorily address all comments and that the manuscript is now suitable for publication in PLOS ONE.

Sincerely,

Amjad Hakami & Sarika Mohan

Department of Health Data Science, Swansea University

Attachment Submitted filename: Response to editor and reviewer.docx

# Editor report: Decision Letter 2

**DOI:** 10.1371/journal.pone.0359436.r006

**Authors:** Muhammad Umer

14 Sep 2026

The perceived facilitators and barriers to the potential adoption of teledentistry among dental care providers in Saudi Arabia: A systematic review.

PONE-D-26-20215R2

Dear Dr. Hakami,

We’re pleased to inform you that your manuscript has been judged scientifically suitable for publication and will be formally accepted for publication once it meets all outstanding technical requirements.

Within one week, you’ll receive an e-mail detailing the required amendments. When these have been addressed, you’ll receive a formal acceptance letter and your manuscript will be scheduled for publication.

An invoice will be generated when your article is formally accepted. Please note, if your institution has a publishing partnership with PLOS and your article meets the relevant criteria, all or part of your publication costs will be covered. Please make sure your user information is up-to-date by logging into Editorial Manager at Editorial Manager® and clicking the ‘Update My Information' link at the top of the page. For questions related to billing, please contact billing support.

If your institution or institutions have a press office, please notify them about your upcoming paper to help maximize its impact. If they’ll be preparing press materials, please inform our press team as soon as possible -- no later than 48 hours after receiving the formal acceptance. Your manuscript will remain under strict press embargo until 2 pm Eastern Time on the date of publication. For more information, please contact onepress@plos.org.

Kind regards,

Muhammad Farooq Umer, PhD Epidemiology and Health Statistics

Academic Editor

PLOS One

Additional Editor Comments (optional):

Reviewers' comments:

# Editor report: Acceptance letter

**DOI:** 10.1371/journal.pone.0359436.r007

**Authors:** Muhammad Umer

PONE-D-26-20215R2

PLOS One

Dear Dr. Hakami,

I'm pleased to inform you that your manuscript has been deemed suitable for publication in PLOS One. Congratulations! Your manuscript is now being handed over to our production team.

At this stage, our production department will prepare your paper for publication. This includes ensuring the following:

* All references, tables, and figures are properly cited

* All relevant supporting information is included in the manuscript submission,

* There are no issues that prevent the paper from being properly typeset

You will receive further instructions from the production team, including instructions on how to review your proof when it is ready. Please keep in mind that we are working through a large volume of accepted articles, so please give us a few days to review your paper and let you know the next and final steps.

Lastly, if your institution or institutions have a press office, please let them know about your upcoming paper now to help maximize its impact. If they'll be preparing press materials, please inform our press team within the next 48 hours. Your manuscript will remain under strict press embargo until 2 pm Eastern Time on the date of publication. For more information, please contact onepress@plos.org.

You will receive an invoice from PLOS for your publication fee after your manuscript has reached the completed accept phase. If you receive an email requesting payment before acceptance or for any other service, this may be a phishing scheme. Learn how to identify phishing emails and protect your accounts at https://explore.plos.org/phishing.

If we can help with anything else, please email us at customercare@plos.org.

Thank you for submitting your work to PLOS One and supporting open access.

Kind regards,

PLOS One Editorial Office Staff

on behalf of

Dr. Muhammad Farooq Umer

Academic Editor

PLOS One
